# The effect of follicular fluid hormones on oocyte recovery after ovarian stimulation: FSH level predicts oocyte recovery

**DOI:** 10.1186/1477-7827-7-35

**Published:** 2009-04-23

**Authors:** Mitchell P Rosen, A Musa Zamah, Shehua Shen, Anthony T Dobson, Charles E McCulloch, Paolo F Rinaudo, Julie D Lamb, Marcelle I Cedars

**Affiliations:** 1 UCSF Medical Center, Department of Obstetrics, Gynecology and Reproductive Sciences, 2356 Sutter Street, 7th Floor, San Francisco, CA 94115, USA

## Abstract

**Background:**

Ovarian stimulation for assisted reproductive technology (ART) overcomes the physiologic process to develop a single dominant follicle. However, following stimulation, egg recovery rates are not 100%. The objective of this study is to determine if the follicular fluid hormonal environment is associated with oocyte recovery.

**Methods:**

This is a prospective study involving patients undergoing ART by standard ovarian stimulation protocols at an urban academic medical center. A total of 143 follicular fluid aspirates were collected from 80 patients. Concentrations of FSH, hCG, estradiol, progesterone, testosterone and prolactin were determined. A multivariable regression analysis was used to investigate the relationship between the follicular fluid hormones and oocyte recovery.

**Results:**

Intrafollicular FSH was significantly associated with oocyte recovery after adjustment for hCG (Adjusted odds ratio (AOR) = 1.21, 95%CI 1.03–1.42). The hCG concentration alone, in the range tested, did not impact the odds of oocyte recovery (AOR = 0.99, 95%CI 0.93–1.07). Estradiol was significantly associated with oocyte recovery (AOR = 0.98, 95% CI 0.96–0.99). After adjustment for progesterone, the strength of association between FSH and oocyte recovery increased (AOR = 1.84, 95%CI 1.45–2.34).

**Conclusion:**

The relationship between FSH and oocyte recovery is significant and appears to work through mechanisms independent of the sex hormones. FSH may be important for the physiologic event of separation of the cumulus-oocyte complex from the follicle wall, thereby influencing oocyte recovery. Current methods for inducing the final stages of oocyte maturation, with hCG administration alone, may not be optimal. Modifications of treatment protocols utilizing additional FSH may enhance oocyte recovery.

## Background

In order to maximize *in vitro *fertilization (IVF) success, ovarian stimulation is performed to generate multiple mature oocytes. This process overrides the physiologic mechanisms that normally select a single dominant follicle. Ovarian stimulation most typically describes the use of exogenous gonadotropins to rescue smaller antral follicles and stimulate the growth of multiple follicles simultaneously. This process yields numerous follicles of varying size with variable rates of oocyte recovery [1].

The regulation of selection, growth, and ovulation of the dominant follicle is a complex process that involves follicle stimulating hormone (FSH), luteinizing hormone (LH) and modulation by an intra-ovarian network of factors [2]. Surges in both FSH and LH precede spontaneous ovulation, characterized by detachment of the cumulus-oocyte complex from the follicular wall and subsequent expulsion from the ovary [3,4]. It is well accepted that LH is obligatory for oocyte nuclear maturation and has a fundamental role in ovulation. However, pure FSH has been shown to stimulate plasminogen activator within the granulosa cells and induce ovulation in hypophysectomized rats [5,6]. Plasminogen activator converts plasminogen to the active protease plasmin, which is presumably involved in dissociating the oocyte from the follicular wall and weakening the wall to facilitate rupture [7,8].

Several studies have suggested follicular diameter correlates with oocyte recovery. We hypothesize oocyte recovery also has a biological basis, which is dependent upon the follicular fluid hormonal milieu. Specifically, we hypothesize that FSH promotes oocyte recovery by stimulating follicle growth while simultaneously initiating follicular hormone production. Then, FSH facilitates the partitioning of the oocyte from the follicular wall either directly or indirectly. Administration of human chorionic gonadotropin (hCG) results in luteinization of the granulosa cells, affecting hormone production and directly or indirectly triggering the detachment of the cumulus-oocyte complex from the follicular wall. The aim of this study was to determine whether any of the intrafollicular hormones we examined would be correlated with oocyte recovery and thereby presumably be important for the still poorly understood process of cumulus-oocyte complex detachment.

## Methods

### Study population

From April 2005 to Sept 2005, 80 patients undergoing ART by standard ovarian stimulation protocols were recruited to collect follicular fluid. Three stimulation protocols were utilized; down regulated (n = 62), microdose flare (n = 10), and antagonist (n = 8). Each patient had 1 or 2 individual follicle aspirates collected at the time of retrieval. A total of 143 follicular fluid aspirates were obtained. One hundred and eleven follicular fluid samples were used to measure hormones. The remaining 32 samples were excluded due to blood contamination or excessive dehydration. This study was approved by the institutional review board at University of California, San Francisco.

### Follicular size measurement and egg retrieval

Individuals were monitored with transvaginal ultrasound (Shimadzu SDU-450XL, Kyoto, Japan) and follicles were measured to obtain a two-dimensional mean diameter. HCG was administrated once two follicles measured 18 mm in mean diameter. A transvaginal ultrasound guided follicular aspiration was conducted 36 hours post hCG administration. Individual follicles of varying size were randomly selected and measured before aspiration. The follicles were categorized into 5 groups according to follicular size at the time of retrieval (the mean of the two-dimension measurements: A, ≥ 18 mm; B, 16–18 mm; C, 13–15 mm; D, 10–12 mm; E, <10 mm).

Prior to aspiration, the collection system (needle and tubing) was flushed to allow for direct correlation of follicular size and fluid volume. The first follicle from either one or both ovaries was aspirated and collected. Each follicle was pierced with a single lumen needle and completely aspirated. The tubing was then flushed and the contents collected in a separate tube to ensure capture of the corresponding oocyte if it was retrieved from the follicle. In order to minimize technical differences, all egg retrievals were performed by one of two physicians. In each patient a maximum of two follicles were aspirated, one from each ovary. Follicles were chosen for our study based on their relative location within the ovary.

For each follicle, the presence or absence of an egg was recorded immediately and the follicular fluid was placed into a 15 ml sterile Falcon conical tube. The follicular size was corrected to the volume of follicular fluid, with a variation between the two methods of measurement averaging 14.6%. The volumes corresponding to each follicle size (<10 mm, <0.5 mL; 10–12 mm, 0.6–1 mL; 13–15 mm, 1.2–2 mL; 16–18 mm, 2.1–3 mL, and >18 mm, >3.1 mL) were similar to those previously described by Wittmaack et al [1]. If the follicular fluid contained blood or was notably dehydrated, the sample was excluded from the study. The follicular fluid was cleared by centrifugation at room temperature for 10 minutes at 1500 × g, aliquoted into 2 ml cryovials, and placed at -80 degrees Celsius for later analysis.

### Follicular fluid hormones

The following hormone concentrations were quantified in duplicate, and measured with commercially available automated chemiluminescent immunoassays on the DPC-Immulite 2000 (Diagnostic Products, Los Angeles, CA): estradiol (E_2_), progesterone (P), total testosterone (T), prolactin (PRL), FSH and hCG. Prior to running samples, follicular fluid assays were validated by dilution testing and confirmation of linearity. These hormones were selected, guided by published literature, because of either their possible or established association with follicular development or to each other. Prior to each test the Immulite 2000 was calibrated with 3 controls of low, medium, and high concentrations of the appropriate hormone. Dilutions were performed prior to measurement of E_2 _(1:1000) and P (1:500) depending on the calibration range. The intraassay coefficient of variations were: E_2 _15%, P 16%, T 13%, PRL 6.8%, and FSH 2.6%. The interassay coefficient of variations were: E_2 _16%, P 16%, T 16.4%, PRL 9.6%, and FSH 5.8%.

### Statistical analysis

Student t-tests were performed on baseline characteristics, and stimulation parameters to compare follicles with oocytes recovered versus not recovered. Repeated measures ANOVA was performed to compare levels of FSH in relation to oocyte recovery. Logistic regression analyses were used to determine if AFC or any of the hormones tested were associated with oocyte recovery after adjusting for follicle size. Then, multivariate models were constructed to determine the independent effects of FSH and hCG by adding one hormone at a time back to the model to assess whether the effect of FSH on oocyte recovery was modified. A final model was constructed adding all hormones that achieved significance to determine the direct effect of FSH on oocyte recovery. Repeated measures linear regression was used to identify the association between follicular fluid gonadotropin and sex steroid concentrations. Both estrogen/progesterone and estrogen/testosterone ratios were also considered as hormone variables. Effect modification of hormones was assessed with an adjustment for follicle size. All analyses were performed using Stata version 9.0 (Stata Corporation, College Station, TX). Tests were declared statistically significant for a two-sided p-value <0.05.

## Results

The characteristics of patients recruited into the study and corresponding stimulation parameters are shown in Table 1. The positive oocyte recovery group included patients where each follicle aspirated contained an egg. If one egg was not recovered from each aspirated follicle they were included in the negative oocyte recovery group. Analyses to determine whether these parameters were associated with oocyte recovery revealed that the total antral follicle count was inversely associated with the odds of oocyte capture (Adjusted odds ratio (AOR) = 0.94, P = 0.016, 95%CI 0.89–0.98). The infertility diagnoses are shown in Table 2. In comparison to an unexplained diagnosis, there is no difference in oocyte recovery with different infertility diagnoses. The average levels of intrafollicular FSH and oocyte recovery separated by stimulation protocol are depicted in Table 3.

**Table 1:** Patient characteristics and stimulation parameters grouped by oocyte recovery

	**100% oocyte recovery** **N = 70**		**< 100% oocyte recovery** **N = 41**				
	**Mean**	**SD**	**Mean**	**SD**	**OR**	**P value**	**CI**
Age	35.53	5.96	34.81	5.17	1.07	0.221	0.95,1.20
Day 3 FSH	6.87	2.75	6.34	1.64	1.17	0.180	0.92,1.48
Start Dose	4.67	1.18	4.46	1.07	1.23	0.298	0.82,1.85
Total Dose	42.00	13.84	38.45	12.61	1.02	0.196	0.98,1.06
Antral follicle count	15.40	8.67	19.35	9.26	0.94	0.016	0.89,0.99
Stimulation days	11.07	1.14	11.14	1.19	0.98	0.939	0.65,1.48
Peak estradiol	2287.64	1252.57	2711.70	1335.41	0.94	0.264	0.95,1.02

**Table 2:** Infertility diagnosis for study patients

**Diagnosis**	**Number (%)**
Unexplained	37 (46)
Diminished Ovarian Reserve	12 (15)
Tubal factor	11 (14)
Ovulatory dysfunction	2 (3)
Other/Combined	18 (22)

χ^2 ^= no difference in oocyte recovery.

**Table 3:** Effect of stimulation protocol on follicular FSH levels and oocyte recovery

**Stimulation Protocol**	**n**	**Avg follicular [FSH]**	**SD [FSH]**	**Proportion oocyte recovery**
Down Regulated	62	4.8 *	3.8	0.63
Microdose Flare	10	12.0 *^ξ^	3.2	0.81
Antagonist	8	7.3 ξ	3.1	0.81

* p <0.001, ^ξ^p < 0.01

**Figure 1:**
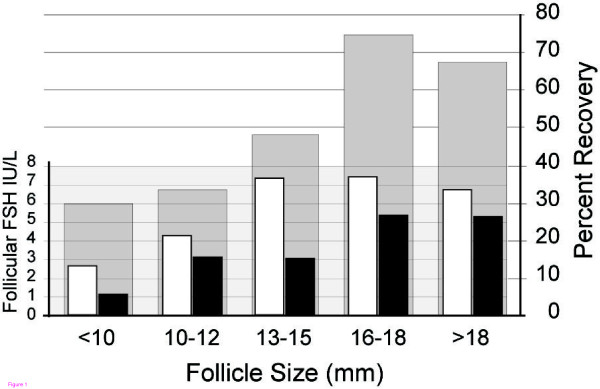
**Follicular FSH level and oocyte recovery**. Rate of oocyte recovery and concentration FSH versus Rate of recovery (gray bars) and FSH concentration in cases of oocyte capture (white bars), and no oocyte capture (black bars). FSH p = 0.007 Oocyte recovery across follicles sizes p <0.001

**Figure 2:**
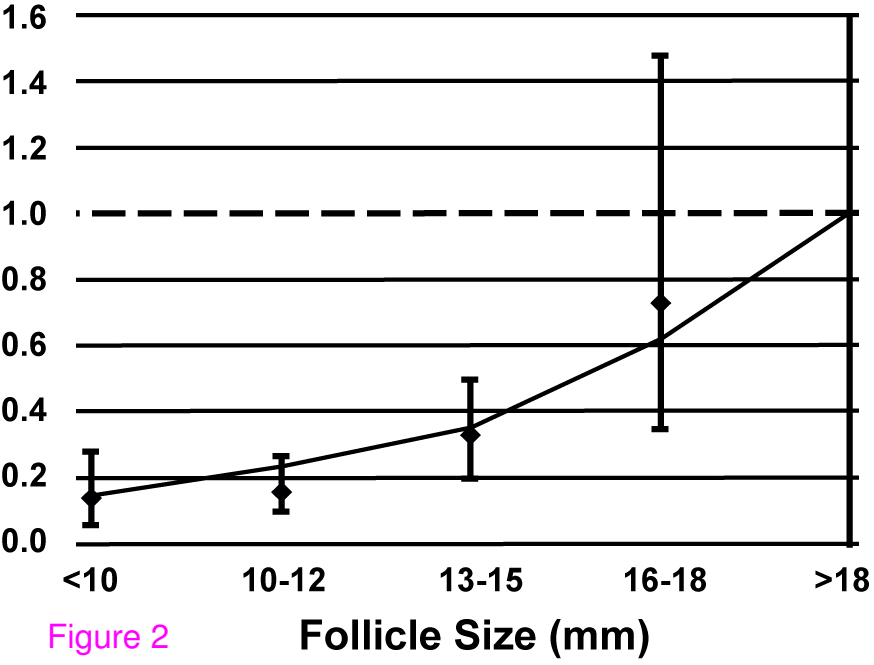
**Odds of oocyte recovery varies with follicle size**. Adjusted odds of oocyte recovery for each follicle size compared to lead follicular group (>18 mm). The relationship of oocyte recovery and follicle size is a monotonic trend (AOR = 0.63, P < 0.001)

The oocyte recovery rate was significantly associated with follicular size. The raw results are illustrated in Figure 1. The odds of oocyte recovery generally decreased with decreasing size (OR: 0.63, P < 0.001, Figure 1, Figure 2). The lead follicular group (Reference, Odds Ratio = 1) is defined as greater than 18 mm in size. The odds of retrieving an egg from follicles of 16–18 mm size was 73% (P = 0.655) compared to the lead follicular group. Oocyte recovery from follicles that were 13–15 mm was 26% (P = 0.020) compared to the lead follicular group, and with smaller sized follicles the chance was further decreased (10–12 mm, 18% (P = 0.001); <10 mm, 14% (P = 0.010)). These findings are consistent with previous work and led us to adjust the potential hormonal effects of oocyte recovery for follicle size [1,9].

The mean follicular fluid hormone concentrations with corresponding standard deviations according to follicular size are summarized in Table 4. The concentrations for FSH (P = 0.01), hCG (P = 0.006), estradiol (P = 0.03), progesterone (P = .0001), and prolactin (P = 0.004) were significantly different between the follicle sizes. The testosterone concentration did not differ (P = 0.16).

**Table 4:** Follicular fluid hormone concentration in cases of oocyte capture (+), and no oocyte capture (-) for each follicle size

**Follicle Size**	**<10 mm**		**10–12 mm**		**13–15 mm**		**16–18 mm**		**>18 mm**	
**Oocyte Captured**	**+**	**-**	**+**	**-**	**+**	**-**	**+**	**-**	**+**	**-**
**(n)**	**(2)**	**(5)**	**(5)**	**(11)**	**(12)**	**(10)**	**(8)**	**(3)**	**(43)**	**(12)**
E2 (ng/mL)	171 ± 100	289 ± 254	322 ± 266	431 ± 278	366 ± 212	442 ± 165	535 ± 308	596 ± 148	498 ± 275	484 ± 219
FSH (IU/L)	2.65 ± 3.2	1.3 ± 1.2	4.2 ± 3.2	3.45 ± 2.7	7.64 ± 3.9 *	3.10 ± 3.1	7.80 ± 4.2	5.73 ± 2.7	6.84 ± 4.5	5.54 ± 3.9
hCG (IU/L)	68.5 ± 46.0	29.8 ± 27.9	105.7 ± 55.3	85.2 ± 51.4	120.3 ± 58.8	96.5 ± 79.2	120.9 ± 91.8	131.0 ± 45.1	78.9 ± 45.3	69.1 ± 21.7
P (μg/mL)	0.2	1.0 ± 0.9	2.4 ± 1.8	2.6 ± 2.1	7.2 ± 4.8	7.6 ± 6.4	12.2 ± 11.8	10.9 ± 2.8	11.8 ± 6.0	13.9 ± 3.2
PRL (ng/mL)	192 ± 156	100 ± 81	323 ± 118	219 ± 95	272 ± 167	369 ± 169	402 ± 289	470 ± 157	352 ± 244	443 ± 201
T (ng/dL)	655 ± 515	337 ± 219	667 ± 529	497 ± 399	385 ± 181	446 ± 148	674 ± 376	749 ± 300	585 ± 353	536 ± 165

Values represent means and standard deviations for each follicular fluid hormone.

* p <0.01

Gonadotropins (FSH, hCG) were significantly associated with the follicular fluid hormone production. Intrafollicular FSH concentration was positively associated with estradiol concentration (P < 0.001) and progesterone concentration (P < 0.001) within the follicle. The FSH concentration did not correlate with testosterone concentration. The hCG concentration correlated with estrogen (P = 0.002) and progesterone concentrations (P < 0.001), but not with the testosterone concentration.

The analyses assessing the impact of each hormone on oocyte recovery adjusting for follicle size are shown (Table 5). Estradiol concentrations were negatively correlated with oocyte recovery, but only slightly (AOR = 0.98, 95% CI 0.96–0.99). The odds of oocyte recovery is 2% less for each 10 ng/dl increase in estradiol.

**Table 5:** The adjusted odds of oocyte recovery for each hormone analyzed individually while adjusted for follicle size

**Follicular Hormone**	**Odds Ratio**	**St. Error**	**P value**	**95% CI**
FSH (IU/L)	1.126	0.071	0.062	0.994–1.275
E2 (ng/mL) §	0.982	0.009	0.035	0.965–0.999
T (ng/dL) §	0.482	0.361	0.330	0.111–2.093
P (μg/mL)	0.946	0.043	0.224	0.864–1.035
PRL (ng/ml)	0.996	0.011	0.725	0.975–1.018
hCG (IU/L)	0.999	0.035	0.985	0.932–1.071
FSH-hCG (IU/L) †§	1.210	0.100	0.021	1.030–1.420

† FSH adjusted for hCG

§ per 10 unit change

The intrafollicular FSH concentration was significantly higher when an oocyte was retrieved than when one was not retrieved (P = 0.007, Figure 1 white versus black bars at each follicle size). In a regression analysis, when adjusted for follicle size, follicular FSH concentrations showed a trend toward positive association with oocyte recovery (AOR = 1.13, 95%CI 0.99–1.27, Table 5). This association between follicular FSH and oocyte recovery was strengthened and statistically significant after adjustment for hCG (AOR = 1.21, 95%CI 1.03–1.42, Table 5); i.e., the odds of retrieving an oocyte increased by 21% for every unit (IU/L) increase of FSH in the follicular fluid when controlled for hCG. In the range tested, hCG concentration alone did not impact the odds of oocyte recovery (AOR = 0.99, 95%CI 0.93–1.07).

When testosterone, estradiol, progesterone, and prolactin were each added to the FSH and hCG model, only progesterone influenced the impact of FSH on the rate of oocyte recovery. It did so by strengthening the FSH and oocyte relationship (AOR = 1.84, 95%CI 1.45–2.34). With progesterone in the model, the odds of oocyte recovery increased by 84% for every IU/L increase in FSH. However, the independent effect of progesterone concentration was negatively associated with oocyte recovery (AOR = 0.79, 95%CI 0.70–0.91); i.e., for every 1 ug/mL increase in progesterone production in the presence of both FSH and hCG, the odds of oocyte recovery decreased by 21%. Follicular estradiol also remained an independent negative predictor of oocyte recovery in the presence of both FSH and hCG (AOR = 0.97, 95%CI 0.95–0.99); i.e., for every 10 ng/dl increase in estradiol, the likelihood of oocyte recovery decreased by 3%. However, estradiol did not modify the FSH effect on oocyte recovery. After adjusting for estradiol and progesterone, while controlling for hCG and follicle size, FSH remained an independent predictor of oocyte recovery (AOR = 1.65, 95%CI 1.22–2.20, Table 6); i.e., the odds of oocyte recovery increased by 65% for every IU/L increase in follicular FSH.

**Table 6:** Multivariate analysis: The independent effects of FSH after adjusting for hCG, progesterone, and estradiol, and controlling for follicle size

**Follicular Hormone**	**Odds Ratio**	**St. Error**	**P>|z|**	**95% Cl**
FSH (IU/L)	1.645	0.244	0.001	1.229–2.202
hCG (IU/L) §	0.999	0.006	0.916	0.985–1.013
E2 (ng/mL) §	0.970	0.015	0.052	0.941–1.000
P (μg/mL)	0.875	0.063	0.062	0.761–1.007

§ per 10 unit change

The association of hormones and oocyte recovery previously observed was similar after adjusting for stimulation protocol. For FSH, after adjusting for follicle size, the effect on oocyte recovery as shown in Table 5 was AOR = 1.13 (95%CI 0.994–1.275). This is essentially the same after also adjusting for stimulation protocol (AOR = 1.16; 95%CI 1.03–1.30). The majority of our follicle aspirates were obtained from down regulated cycles, and even solely within this group the association of FSH and oocyte recovery was similar (AOR = 1.22; 95%CI 1.07–1.39).

## Discussion

While the decision to go to egg retrieval is based largely upon the size of the lead follicular group, an attempt is usually made to retrieve oocytes regardless of follicular size. Several studies have suggested that the chance of recovering an oocyte decreases with smaller follicular size, and our findings are consistent with this [1,9]. A recent publication from our group has also shown that the odds of oocyte nuclear maturation and normal fertilization are decreased in oocytes derived from smaller follicles [10]. Developmentally, each follicle contains an oocyte. Yet there are reports of no oocyte recovery from IVF cycles involving multiple mature sized follicles, and in virtually all ovarian stimulation cycles the rate of oocyte recovery is less than 100% relative to the number of observed follicles [11]. Therefore, assuming a similar aspiration technique is used, one reason why no egg is retrieved from certain follicles is that the cumulus-oocyte complex remains attached to the follicular wall during aspiration. Breaking this connection is a vital process for normal ovulation, and aspirated follicles where no egg is retrieved may allow elucidation of this process. We performed a repeated measures logistic regression analysis examining the oocyte recovery rates at different follicle sizes. The results demonstrate a significant correlation with the odds of retrieving an oocyte decreasing as the follicle size decreases (Figure 2).

Interestingly, we found that FSH levels were higher in follicles in which an oocyte was recovered, and this was significant after adjusting for the type of ovarian stimulation protocol. An adjustment for stimulation was made due to different management schemes used in the protocols, as commonly we step-down with down regulated protocols resulting in potential differences in follicular fluid FSH levels (Table 3). We could not adequately compare the oocyte recovery between stimulation protocols due to sample size. However, the fact that follicle number is inversely predictive of oocyte recovery is consistent with follicular fluid FSH being associated with oocyte recovery, because down regulated cycles (which had on average lower follicular FSH) are typically prescribed in women with higher follicle numbers.

While FSH is an important contributor to oocyte recovery, it is only significant after adjustment for hCG (Table 5). This is an interesting finding, since in spontaneous ovulation there is a surge in both FSH and LH. Whether the FSH and LH surges have overlapping, complementary or redundant functions is unknown. In high concentrations, FSH has been shown to induce ovulation by itself in certain animal models. In hypophysectomized rats, pure recombinant FSH as a large bolus has been shown to induce ovulation, although the dose of FSH required was larger than the dose of hCG needed to promote ovulation [5,6]. In rhesus monkeys who were down regulated with GnRH agonist and undergoing IVF, recombinant FSH was shown to recapitulate some but not all of the characteristics of recombinant hCG. Specifically, r-hFSH was equivalent to r-hCG for the reinitiation of oocyte meiosis, fertilization and granulosa cell luteinization, but a midcycle FSH surge did not sustain normal luteal function [12]. A role for FSH is supported by *in vitro *experiments, where both FSH and LH can promote plasminogen activator activity in cultured granulosa cells [6,13]. These results also have biochemical plausibility as both FSH and LH receptors primarily mediate downstream signaling events through activation of the stimulatory G protein Gs with resultant increases in intracellular cyclic AMP. Differences in signals from G protein coupled receptors (GPCRs) that activate the same G protein are presumably due to either different characteristics of cyclic AMP fluxes within the cell or the activation of alternate signaling pathways not mediated specifically by G protein interactions [14]. Granulosa cell responsiveness to LH may be dependent upon whether the cells have been previously exposed to other hormones [15]. Reich et al, showed in a mouse model that the potency of LH is enhanced in the presence of estradiol [6]. Evidence in humans, at least in the natural cycle, also suggests that plasminogen activity is positively correlated with estradiol levels [16]. However, this observation was not seen in the setting of ovarian stimulation and supraphysiologic estradiol levels, and it was suggested that a subtle balance exists between granulosa cell secretion of plasminogen activator and steroids that is disturbed during controlled ovarian hyper-stimulation [16].

Although the precise functions of FSH and LH in ovulation are not completely known, in ovulation induction cycles, hCG, which activates LH receptors, is administered to promote the periovulatory events and induce ovulation. In the human menstrual cycle, there is a mid-cycle surge in both FSH and LH secretion in the periovulatory period. Presumably these hormones act synergistically to promote the optimum environment for final follicle maturation and ovulation. In our study, neither hCG nor FSH alone was significantly associated with oocyte recovery when analyzed independently. The excess amount of hCG administered and available at the follicular level may account for the absence of an association. Interestingly, when both FSH and hCG were added to the model, only FSH became an independent predictor of oocyte recovery during follicular aspiration. Although more studies are needed, it is possible that FSH participates in the ovulation process either directly, by stimulating plasminogen activity, or indirectly by enhancing the responsiveness to hCG (LH) via modulation of the follicular environment (i.e. the hormonal milieu).

The association between higher FSH concentrations within follicles from which an oocyte was retrieved may suggest a role for increased vascularity around these follicles, since the follicular FSH is ultimately serum-derived [12]. Following ovarian stimulation, follicles have different levels of oxygen tension [17]. It is possible that while the amount of hCG is in excess, the FSH concentration may be at a threshold and would be vulnerable to changes in vascularity. However, if vascularity completely explained the correlation between FSH and oocyte recovery, other serum hormones impacted by changes in vascularity would be expected to correlate with oocyte recovery as well. Our data suggest there is an effect of FSH beyond that due to vascularity alone since prolactin, which is transported via the serum, has no association with oocyte recovery. This implies that other mechanisms may regulate FSH levels within the follicle. For example, the healthier follicles may be secreting factors such as VEGF, which is known to increase capillary permeability and thereby allow for enhanced diffusion of larger molecules such as FSH [18,19]. Another possibility is there may be sequestration or preferential uptake of FSH in healthier metabolically active follicles.

Our analysis shows estradiol levels are inversely associated with oocyte recovery when measured alone or after adjusting for hCG and FSH. Physiologically, follicular estradiol is derived from androgen precursors and its production is influenced by FSH-induced aromatase activity [2]. In our study, while the association of FSH and estradiol was significant, the ratio of estradiol/testosterone was not predictive of oocyte recovery, nor did it modify the effect of FSH. Estradiol production normally decreases after the midcycle surge of LH as a result of luteinization [2]. It is possible that high concentrations of estradiol are a surrogate marker for inadequate response of the granulosa cells to hCG and thus indicate decreased ability of the oocyte to detach from the follicular wall.

A limitation of this study is that these findings are associative and cause and effect cannot be determined. Additionally, the relationship between plasma FSH and follicular fluid FSH at the time of hCG trigger or retrieval would be interesting to explore. Our data set is limited to plasma FSH levels obtained when the patient is not receiving exogenous gonadotropins and therefore we cannot explore this relationship. Another limitation is the numbers of patients analyzed within the subgroups of follicle size are limited (Table 4). However, the results have biological plausibility, and provide a valid answer to the clinical question of whether there are any biochemical predictors of oocyte recovery.

The relationship between FSH and oocyte recovery was independent of the sex steroids, but the association was strengthened in the presence of progesterone which itself had a negative effect on oocyte recovery (Table 6). This suggests that oocyte recovery could be improved if we can devise ways to increase follicular FSH without increasing progesterone. HCG is primarily responsible for luteinization of granulosa cells and subsequent progesterone production. High progesterone levels in this context may be an indicator of excess hCG or relative lack of FSH. Based on these data, and the hypothesis that a mid-cycle bolus of FSH will enhance oocyte recovery by promoting cumulus-oocyte complex release from the follicle wall, we have instituted a randomized, controlled study to evaluate the effects of FSH administration at the time of the hCG trigger during IVF. More studies are needed to determine the biological basis for the impact of FSH on oocyte recovery and to determine whether modifying gonadotropin administration can improve oocyte recovery rates.

## Competing interests

The authors declare that they have no competing interests.

## Authors' contributions

MPR conceived of the study, and participated in its design and coordination and helped to draft the manuscript. AMZ participated in data analysis and interpretation and helped to draft the manuscript. SS participated in study design and data collection and ran immunoassays. ATD participated in study design and coordination and helped to draft the manuscript. CEM performed the statistical analyses and helped to draft the manuscript. PR participated in study design and data analysis and interpretation. JDL helped to draft the manuscript. MIC participated in study design and coordination and helped to draft the manuscript. All authors read and approved the final manuscript.

## References

[B1] Wittmaack FM, Kreger DO, Blasco L, Tureck RW, Mastroianni L, Lessey BA (1994). Effect of follicular size on oocyte retrieval, fertilization, cleavage, and embryo quality in in vitro fertilization cycles: a 6-year data collection. Fertil Steril.

[B2] Gougeon A (1996). Regulation of ovarian follicular development in primates: facts and hypotheses. Endocr Rev.

[B3] Hoff JD, Quigley ME, Yen SS (1983). Hormonal dynamics at midcycle: a reevaluation. J Clin Endocrinol Metab.

[B4] Thorneycroft IH, Mishell DR, Stone SC, Kharma KM, Nakamura RM (1971). The relation of serum 17-hydroxyprogesterone and estradiol-17-beta levels during the human menstrual cycle. Am J Obstet Gynecol.

[B5] Galway AB, Lapolt PS, Tsafriri A, Dargan CM, Boime I, Hsueh AJ (1990). Recombinant follicle-stimulating hormone induces ovulation and tissue plasminogen activator expression in hypophysectomized rats. Endocrinology.

[B6] Reich R, Miskin R, Tsafriri A (1985). Follicular plasminogen activator: involvement in ovulation. Endocrinology.

[B7] Morioka N, Zhu C, Brannstrom M, Woessner JF, LeMaire WJ (1989). Mechanism of mammalian ovulation. Prog Clin Biol Res.

[B8] Strickland S, Beers WH (1976). Studies on the role of plasminogen activator in ovulation. In vitro response of granulosa cells to gonadotropins, cyclic nucleotides, and prostaglandins. J Biol Chem.

[B9] Scott RT, Hofmann GE, Muasher SJ, Acosta AA, Kreiner DK, Rosenwaks Z (1989). Correlation of follicular diameter with oocyte recovery and maturity at the time of transvaginal follicular aspiration. J In Vitro Fert Embryo Transf.

[B10] Rosen MP, Shen S, Dobson AT, Rinaudo PF, McCulloch CE, Cedars MI (2008). A quantitative assessment of follicle size on oocyte developmental competence. Fertil Steril.

[B11] Stevenson TL, Lashen H (2008). Empty follicle syndrome: the reality of a controversial syndrome, a systematic review. Fertil Steril.

[B12] Christenson LK, Stouffer RL (1997). Follicle-stimulating hormone and luteinizing hormone/chorionic gonadotropin stimulation of vascular endothelial growth factor production by macaque granulosa cells from pre- and periovulatory follicles. J Clin Endocrinol Metab.

[B13] Wang C, Leung A (1983). Gonadotropins regulate plasminogen activator production by rat granulosa cells. Endocrinology.

[B14] Shenoy SK, Lefkowitz RJ (2005). Seven-transmembrane receptor signaling through beta-arrestin. Sci STKE.

[B15] Canipari R, Strickland S (1986). Studies on the hormonal regulation of plasminogen activator production in the rat ovary. Endocrinology.

[B16] Weimer SL, Campeau JD, Marrs RP, Dizerega GS (1984). Alteration of human follicular fluid plasminogen activator activity by ovarian hyperstimulation. J In Vitro Fert Embryo Transf.

[B17] Van Blerkom J, Antczak M, Schrader R (1997). The developmental potential of the human oocyte is related to the dissolved oxygen content of follicular fluid: association with vascular endothelial growth factor levels and perifollicular blood flow characteristics. Hum Reprod.

[B18] Gutman G, Barak V, Maslovitz S, Amit A, Lessing JB, Geva E (2008). Regulation of vascular endothelial growth factor-A and its soluble receptor sFlt-1 by luteinizing hormone in vivo: implication for ovarian follicle angiogenesis. Fertil Steril.

[B19] Monteleone P, Giovanni Artini P, Simi G, Casarosa E, Cela V, Genazzani AR (2008). Follicular fluid VEGF levels directly correlate with perifollicular blood flow in normoresponder patients undergoing IVF. J Assist Reprod Genet.

